# Clinical-grade human dental pulp stem cells suppressed the activation of osteoarthritic macrophages and attenuated cartilaginous damage in a rabbit osteoarthritis model

**DOI:** 10.1186/s13287-021-02353-2

**Published:** 2021-05-01

**Authors:** Pei-Lin Li, Yu-Xing Wang, Zhi-Dong Zhao, Zhi-Ling Li, Jia-Wu Liang, Qian Wang, Bo-Feng Yin, Rui-Cong Hao, Meng-Yue Han, Li Ding, Chu-Tse Wu, Heng Zhu

**Affiliations:** 1 Beijing Institute of Radiation Medicine, Road Taiping 27, Beijing, 100850 P. R. China; 2 Beijing Key Laboratory for Radiobiology, Beijing Institute of Radiation Medicine, Beijing, 100850 P. R. China; 3 The Affiliated People’s Hospital of Jiangsu University, Zhenjiang, Jiangsu Province, People’s Republic of China; 4 People’s Liberation Army General Hospital, Road Fuxing 28, Beijing, 100853 P. R. China; 5 Graduate School of Anhui Medical University, 81 Meishan Road, Shu Shan Qu, Hefei, 230032 Anhui P. R. China; 6 Medical Center of Air Forces, PLA, Road Fucheng 30, Beijing, 100142 P. R. China

**Keywords:** Human dental pulp stem cells, Osteoarthritic macrophages, Cartilaginous damage, Osteoarthritis

## Abstract

**Background:**

Although increasing evidence has demonstrated that human dental pulp stem cells (hDPSCs) are efficacious for the clinical treatment of skeletal disorders, the underlying mechanisms remain incompletely understood. Osteoarthritis (OA) is one of the most common degenerative disorders in joints and is characterized by gradual and irreversible cartilaginous tissue damage. Notably, immune factors were newly identified to be closely related to OA development. In this study, we explored the modulatory effects of clinical-grade hDPSCs on osteoarthritic macrophages and their protective effects on cartilaginous tissues in OA joints.

**Methods:**

The cell morphology, immunophenotype, and inflammatory factor expression of osteoarthritic macrophages were explored by phase contrast microscope, transmission electron microscopy, immunostaining, flow cytometry, quantitative polymerase chain reaction, and enzyme linked immunosorbent assay, respectively. Additionally, the factors and signaling pathways that suppressed macrophage activation by hDPSCs were determined by enzyme-linked immunosorbent assay and western-blotting. Furthermore, hDPSCs were administered to a rabbit knee OA model via intra-articular injection. Macrophage activation in vivo and cartilaginous tissue damage were also evaluated by pathological analysis.

**Results:**

We found that hDPSCs markedly inhibited osteoarthritic macrophage activation in vitro. The cell morphology, immunophenotype, and inflammatory factor expression of osteoarthritic macrophages changed into less inflammatory status in the presence of hDPSCs. Mechanistically, we observed that hDPSC-derived hepatocyte growth factor and transforming growth factor β1 mediated the suppressive effects on osteoarthritic macrophages. Moreover, phosphorylation of MAPK pathway proteins contributed to osteoarthritic macrophage activation, and hDPSCs suppressed their activation by partially inactivating those pathways. Most importantly, injected hDPSCs inhibited macrophage activation in osteochondral tissues in a rabbit knee OA model in vivo. Further histological analysis showed that hDPSCs alleviated cartilaginous damage to knee joints.

**Conclusions:**

In summary, our findings reveal a novel function for hDPSCs in suppressing osteoarthritic macrophages and suggest that macrophages are efficient cellular targets of hDPSCs for alleviation of cartilaginous damage in OA.

**Graphical abstract:**

hDPSCs treat OA via an osteoarthritic macrophages-dependent mechanisms. hDPSCs suppress the activation of osteoarthritic macrophages in vitro and in vivo and alleviate cartilaginous lesions in OA models. 
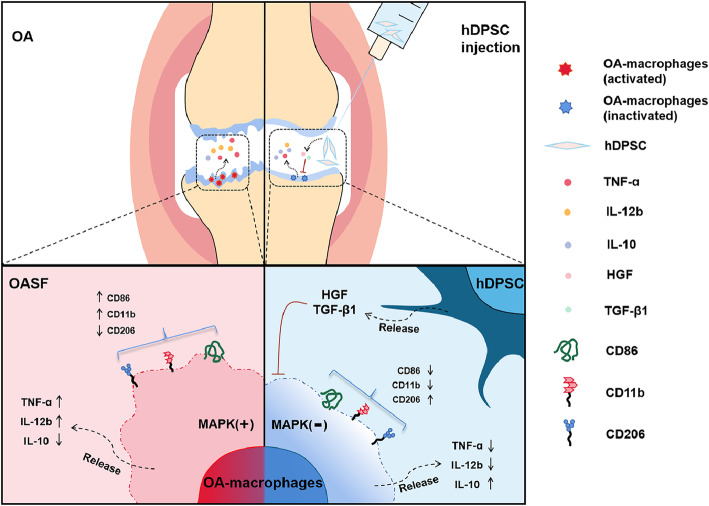

**Supplementary Information:**

The online version contains supplementary material available at 10.1186/s13287-021-02353-2.

## Introduction

Osteoarthritis (OA) is a disease of an entire synovial joint characterized by degradation of joint cartilaginous tissues, including articular cartilage and meniscus, and clinical symptoms, such as joint pain and disability [1, 2]. Although a number of therapeutic strategies have been developed to alleviate tissue damage in OA, including lifestyle improvement, pharmaceutical management, and arthroscopic debridement, currently, there are few curable treatments available until the end stage of the disease necessitates joint replacement [3, 4]. Accumulated studies prove that complex crosstalk of genetic, biochemical, biomechanical, and metabolic factors is involved in OA development [5]. Notably, increasing evidence has demonstrated that activation of immune cells and subsequent inflammatory responses contribute to tissue injuries in OA [6–8].

Macrophages are hematopoietic stem cell-derived immune cells and have now been shown to play a prominent role in the progression of OA. Increasing evidence has demonstrated that overactivated macrophages promote the inflammatory microenvironment, increase the secretion of matrix metalloproteinases, inhibit the proliferation and viability of joint resident stem cells, and prevent cartilage repair [9–11]. Haraden et al. reported that osteoarthritic macrophages highly expressed costimulatory factors such CD80 and CD86, which further initiated the adaptive immune response and induced expanded tissue injury [11, 12]. In addition, Fahy et al. stated that the osteoarthritic macrophage-associated cytokines interleukin-12 (IL-12) and tumor necrosis factor-α (TNF-α) induced destructive processes of articular cartilage by suppressing collagen type II and aggrecan synthesis [13]. Furthermore, osteoarthritic macrophages were shown to negatively affect the chondrogenesis of stem cells in OA [13, 14]. Thus, these data suggest the therapeutic potential of modulating macrophages to protect cartilaginous tissues in OA.

Human dental pulp stem cells (hDPSCs) are isolated from dental pulp and express a low level of costimulatory molecules, which indicates that they are at a low risk of triggering immune rejection against themselves [15, 16]. Under certain conditions, hDPSCs are capable of differentiating into multiple types of tissue cells that contribute to skeletal regeneration. Further, previous studies have suggested that hDPSCs exhibit enhanced immunomodulatory properties compared with traditional mesenchymal stem cells (MSCs) by secreting multiple immune factors including hepatocyte growth factor (HGF) and transforming growth factor beta 1 (TGFβ1) [17, 18]. Moreover, preparation of hDPSCs is less invasive compared to that of other MSCs. Given their low immunogenicity, strong tissue repair capacity, immunomodulatory properties, and convenient availability, hDPSCs may be an efficient tool for alleviating cartilaginous tissue injuries in OA by targeting macrophages.

In recent decades, we have been pursuing basic research and translational applications of stem cell-based therapy for the treatment of severe diseases [19–26]. Notably, we explored the therapeutic effects of hDPSCs on several skeletal disorders, including rheumatoid arthritis, osteoporosis, and tooth loss [27–29]. These studies demonstrated that transfusing hDPSCs yields promising results in maintaining skeletal homeostasis. In addition, we launched clinical trials using clinical-grade hDPSCs to treat patients with severe COVID-19 [30].

In the current study, we explored the effects of clinical-grade hDPSCs on osteoarthritic macrophages in vitro and in vivo. Furthermore, the underlying cellular and molecular mechanisms regarding the capacity of hDPSCs to regulate osteoarthritic macrophages were investigated. Moreover, the protective effects of hDPSCs on articular cartilage in a knee OA model were evaluated.

## Materials and methods

### Animals

Normal inbred male New Zealand White rabbits (weighing 3.0–3.5 kg, *n*=21) were purchased from the Laboratory Animal Center of the Academy of Military Medical Sciences of China (Beijing). All of the animal experiments were performed in accordance with the Academy of Military Medical Sciences Guide for Laboratory Animals.

### hDPSC preparation

Clinical-grade hDPSCs were obtained from Beijing SH Biotechnology (http://www.bjshbio.com/). The hDPSCs were prepared as previously described with written consent [28]. In brief, hDPSCs were isolated and cultured in a GMP-compliant facility following ISO 8 clean room standards. The pulp tissues were separated from the root and crown, digested by animal origin-free collagenase, and cultured by xenobiotic-free cell culture reagents (donors=4). The immunophenotype and the multiple-differentiation of the hDPSCs were evaluated as previously reported. Briefly, Passage 3–4 hDPSCs which showed an immunophenotype similar to bone marrow MSCs (BMMSCs) and multiple-differentiation capacities were used for in vivo and in vitro experiments unless otherwise described.

### Human osteoarthritic macrophage generation

Synovial fluid from OA patients (OASF) is a potent inflammatory mediator that includes numerous inflammatory cytokines and has been reported to promote osteochondral lesions in osteoarthritis [20, 31, 32]. In the current study, OASF samples were independent combinations from 10 OA patients and were used to mimic the joint microenvironments of OA and to cultivate osteoarthritic macrophages. Human OASF was harvested from the knee joints of 10 patients with OA with previous protocols [20] (Tab. S1). Ethics approval was obtained from the PLA General Hospital Research Ethics Committee, and participant consent was acquired prior to sample collection.

Human peripheral blood mononuclear cells were isolated by Ficoll-Paque (1.077 g/mL; Invitrogen) density gradient centrifugation from healthy volunteers (*n*=6), in which we have obtained the informed consents from the participants and it has been approved by the Research Ethics Committee of PLA Medical Center of Air Forces. CD14^+^ monocytes were further harvested from PBMCs using the MACS Monocyte Isolation Kit (Miltenyi Biotec, Bergisch Gladbach, Germany). To generate osteoarthritic macrophages, 2×10^6^ monocytes (more than 85% CD14^+^) were cultured in 3 ml of α-MEM supplemented with 10% fetal bovine serum in the presence of human recombinant macrophage-stimulating factor (M-CSF, 50 ng/ml) for 3 days and with the addition of 20% vol/vol human OASF for another 3 days.

To investigate the underlying mechanisms of hDPSCs on osteoarthritic macrophages, hDPSCs were directly co-cultured with osteoarthritic macrophages or cocultured with osteoarthritic macrophages in a Transwell coculture system at graded cell ratios. The hDPSCs were grown in lower compartment of Transwell coculture system at a density of 2×10^5^/cm^2^ while osteoarthritic macrophages were seeded in upper compartment. The hDPSCs and osteoarthritic macrophages were cocultured at different hDPSCs to macropohage ratios (1:100, 1:50, and 1:10) for 48 h. In some experiments, anti-human HGF (200 ng/ml) and anti-human TGFβ1 (200 ng/ml) neutralizing antibody were added into the hDPSC to macrophage Transwell culture system. All experiments above have been performed in triplicates for three times unless other described.

### Cell morphology and ultrastructure of human osteoarthritic macrophages

A total of 2×10^6^ osteoarthritic macrophages were directly cocultured with 2×10^6^ hDPSCs in 6-well plates for 48 h. The cell morphology of the osteoarthritic macrophages or hDPSCs-osteoarthritic macrophages was observed under a phase contrast microscope (Nikon TE2000-U). For transmission electron microscopy ultrastructural observations (TEM, HITACHI, Tokyo, Japan), osteoarthritic macrophages or hDPSC-osteoarthritic macrophages were collected and fixed for 4 h at 4 °C in 5% glutaraldehyde, washed 3 times in 0.1 mol/L phosphate-buffered saline (PBS), postfixed for 2 h at 4 °C in 2% osmium tetroxide, dehydrated in a graded series of ethanol, embedded in Epon 812, cut into ultrathin sections (75 nm), and then stained with uranyl acetate and lead citrate. The sections were then viewed and recorded with a HITACHI H-600 electron microscope at 80 kV.

### Immunophenotyping and immunostaining of osteoarthritic macrophages

To detect the effect of hDPSCs on the osteoarthritic macrophage immunophenotype in vitro, osteoarthritic macrophages were directly cocultured with hDPSCs at different ratios for 48 h. The hDPSC to osteoarthritic macrophage ratios were 1:100, 1:50, and 1:10. The macrophages were stained with FITC-, PE-, or allophycocyanin (APC)-conjugated monoclonal antibodies against human CD11b (Clone ICRF44, BD, 20μl/sample), CD86 (Clone L307.4, BD, 5μl/sample), and CD206 (Clone 19.2, BD, 20μl/sample) according to the manufacturer’s protocol. Unstained cells were used as control. Events were collected by flow cytometry with a FACSCalibur system (Bectone Dickinson). The unstained cells were used as negative controls and the gating strategy was based on the forward scatter and side scatter of the samples, and combined with the previous study experiences. The flow analysis has been performed by using Flowjo 8.0.

In addition, macrophages and hDPSC-macrophages were stained with CD11b (Clone M1/70, Abcam, 1:500 dilution) and CD68 (Clone KP1, BD, 1:500) antibodies and fluorescent secondary antibodies and observed under a fluorescence microscope as previously reported. In brief, OA-macrophage on coverslips (ZSGB-bio, Beijing) were washed 3 times with PBS before being fixed in 4% paraformaldehyde for 15 min. Cells were further incubated with PBT1 (PBS/1% bovine serum albumin/5% normal goat serum/0.1% Triton X-100) at room temperature for 60 min and then with CD11b or CD68 antibodies overnight respectively. Then, the cells were washed by PBT1 and PBT2 (PBS/0.1% bovine serum albumin/0.1% Triton X-100) for two times. Moreover, the cells were incubated with secondary antibodies at 1:250 for 2 h before they were washed twice in PBT2, followed by 3 washes with PBS. The cells were further stained with DAPI. The data were collected by using fluorescent microscope (Leica, Germany).

### Immune factor determination by real-time PCR and ELISA

To assess the effect of hDPSCs on immune cytokine expression in macrophages in vitro, hDPSCs were cocultured with osteoarthritic macrophages in Transwell system at different ratios for 48 h. The hDPSC to macrophage ratios were 1:100, 1:50, and 1:10. The culture medium was removed and the macrophages were harvested by EDTA-2Na (2mm) with cold PBS. The macrophages were further washed by PBS for two times before the RNA extraction. Total RNA was extracted with TRIzol reagent (Invitrogen) and reverse transcribed using the mRNA Selective PCR kit (TaKaRa). Human TNF-α, IL-10, and IL-12b cDNAs were amplified by real-time PCR using the SYBR Green PCR kit (Sigma) and a 7500 Real-Time PCR Detection System (Applied Biosystems, ABI). The mRNA levels were normalized to the value of glyceraldehyde-3-phosphate dehydrogenase (GAPDH). To compare the expression of human TNF-α, IL-10, and IL-12b transcripts between treated and untreated samples by relative quantification (2^−△△CT^). Take cDNA for RT-qPCR reaction, add sample according to the ratio in Table S2, immediately centrifuge and mix well, and amplify according to the conditions in Table S3. The Melt Curve Stage is automatically set by the instrument, as shown in Table S4. The primer sequences used for real-time PCR are shown in Table S5 [33–35].

Meanwhile, the culture supernatants were harvested from hDPSC-treated osteoarthritic macrophages. In brief, the upper compartments with the osteoarthritic macrophages of hDPSC-macrophage Transwell culture systems were transferred to a new culture plate and the osteoarthritic macrophages were cultured for 24 h in the absence of hDPSCs. The culture medium of hDPSC-treated osteoarthritic macrophages were collected by centrifugation and filtered to deplete the cellular components. Human TNF-α, IL-10, and IL-12b concentrations in the supernatants were determined according to the reagent protocols of the quantitative determination kit (R&D Systems, Minneapolis). Optical density was read at 450 nm by using a Microplate reader (Thermo Scientific Varioskan Flash). For each experimental culture well, duplicate ELISA readings were obtained.

### Western blotting

To explore the signaling pathways involved in the effects of hDPSCs on osteoarthritic macrophages, the conditional medium (2×10^5^/cm^2^ hDPSCs in 3 ml for 72 h) was added into the culture system of osteoarthritic macrophage at a ratio of 10% (vol/vol). The specific inhibitors of the P38/MAPK, ERK/MAPK, and JNK/SAPK pathways (10μM) were added to osteoarthritic macrophage culture systems to further reveal the molecular mechanisms, respectively. Osteoarthritic macrophages were collected at 0, 5, 10, 30, and 60 min. Protein lysis buffer (BioRad, Hercules, CA) was added, and the thawed lysates were vortexed and centrifuged. Proteins were separated by 10% sodium dodecyl sulfate-polyacrylamide gel electrophoresis and transferred onto nitrocellulose membranes. The membranes were blocked by incubation with 5% wt/vol nonfat dry milk. The membranes were then incubated with anti-p38, anti-phospho-38 (P-p38), anti-ERK, anti-phospho-ERK (P-ERK), anti-JNK, anti-phospho-JNK (P-JNK), and GADPH antibodies (Cell Signaling Technology) at a dilution of 1:1000 (vol/vol) overnight at 4°C. After incubation, the membranes were washed in Tris-buffered saline with Tween-20 (TBST). Horseradish peroxidase-conjugated secondary antibodies at a dilution of 1:2000 (vol/vol) were added to the membranes in 5% nonfat dry milk in TBST. The Fiji software was used to perform grayscale analysis of bands for quantitative comparisons.

### Transplantation of hDPSCs to a rabbit knee OA model

In the current study, a rabbit OA model was induced in both knees of each of 21 rabbits by anterior cruciate ligament sectioning. All rabbits were randomly allocated to 3 groups, as shown in Table 1. Briefly, each rabbit was anesthetized with intramuscular injection of xylazine (5 mg/kg) and ketamine (35 mg/kg), and anesthesia was maintained by additional bolus of ketamine (20mg/kg). The surgery was performed under general anesthesia. After removing hair around the knees, the skin was disinfected with povidone-iodine and 70% ethanol. The medial skin of the knee joint, fascia, and joint capsule were cut. The anterior cruciate ligament (ACL) was exposed, and the medial parenchyma of the ACL and the meniscus ligament were completely cut. Then, the joint capsule, subcutaneous tissue and skin were sutured and disinfected with povidone-iodine. Intramuscular penicillin injections were administered to each rabbit to prevent infection. The condition of the surgical incision was checked to see if there is any redness, swelling, and exudation. The general conditions of the rabbit appetite and weight were monitored. To inject hDPSCs, the rabbits were anesthetized as described above. The hDPSCs were suspended in physiological saline solution. hDPSCs (2×10^5^/knee joint and 1×10^6^/knee joint, in 250μl/knee joint, respectively) were intraarticularly injected into the OA rabbit model 1 week post operation. The control group received physiological saline solution (250μl/knee joint) alone to account for the effect of injection. All animals were sacrificed 10 weeks after surgery for further evaluation.

**Table 1:** Experimental groups of the in vivo study

Groups	Rabbit number	Treatment
OA group	7	Anterior cruciate ligament sectioning
OA+hDPSCs group (2×10^5^/knee joint)	7	Anterior cruciate ligament sectioning + hDPSCs (2×10^5^/knee joint) intraarticular injection
OA+hDPSCs group (1×10^6^/knee joint)	7	Anterior cruciate ligament sectioning + hDPSCs (1×10^6^/knee joint) intraarticular injection

*OA* osteoarthritis, *hDPSCs* human dental pulp stem cells

### Gross observation and pathological analysis

The dissected proximal parts of the tibia were evaluated following protocols recommended by OARSI for the rabbit OA models [31, 32]. To perform histopathological analysis, all specimens were fixed in 4% paraformaldehyde for 7 days, decalcified in 10% EDTA solution for 30 days, embedded in paraffin, sectioned into 6-μm slices, and stained by HE staining. The cartilaginous matrix distribution was evaluated by Toluidine blue and Safranin-O/Fast Green. The microscopic assessments of the knee tissues were conducted based on the data of HE and Safranin-O/Fast Green staining followed the scoring system recommended by OARSI for the rabbit OA models [31, 32]. The expression of collagen type II in the knee tissues was determined by immunohistochemical analysis. Briefly, sections were treated with methanol to H2O2 (49:1) for 30 min, with 1 mg/ml hyaluronidase in PBS pH 6.0, for 30 min at 37 C, and goat serum for 1 h. An anti-type II collagens (Abcam) were added at a dilution of 1:500 (vol/vol) for 1 h at room temperature. Biotinylated secondary antibodies and streptavidin-peroxidase followed by incubation with AEC chromogen substrate (Sigma, Germany) was used. Stained slides were observed at different magnifications and images were acquired with the microscope (Leica, Germany). The CD11b^+^CD68^+^ macrophages in specimens were shown by in situ immunofluorescence. For immunofluorescent analysis, the procedures are similar to that of cellular immunofluorescence. Anti-CD11b and anti-CD68 antibodies were incubated at a dilution of 1:500 (vol/vol). The data were collected by using fluorescent microscope (Leica, Germany).

### Statistical analysis

**Fig. 1:**
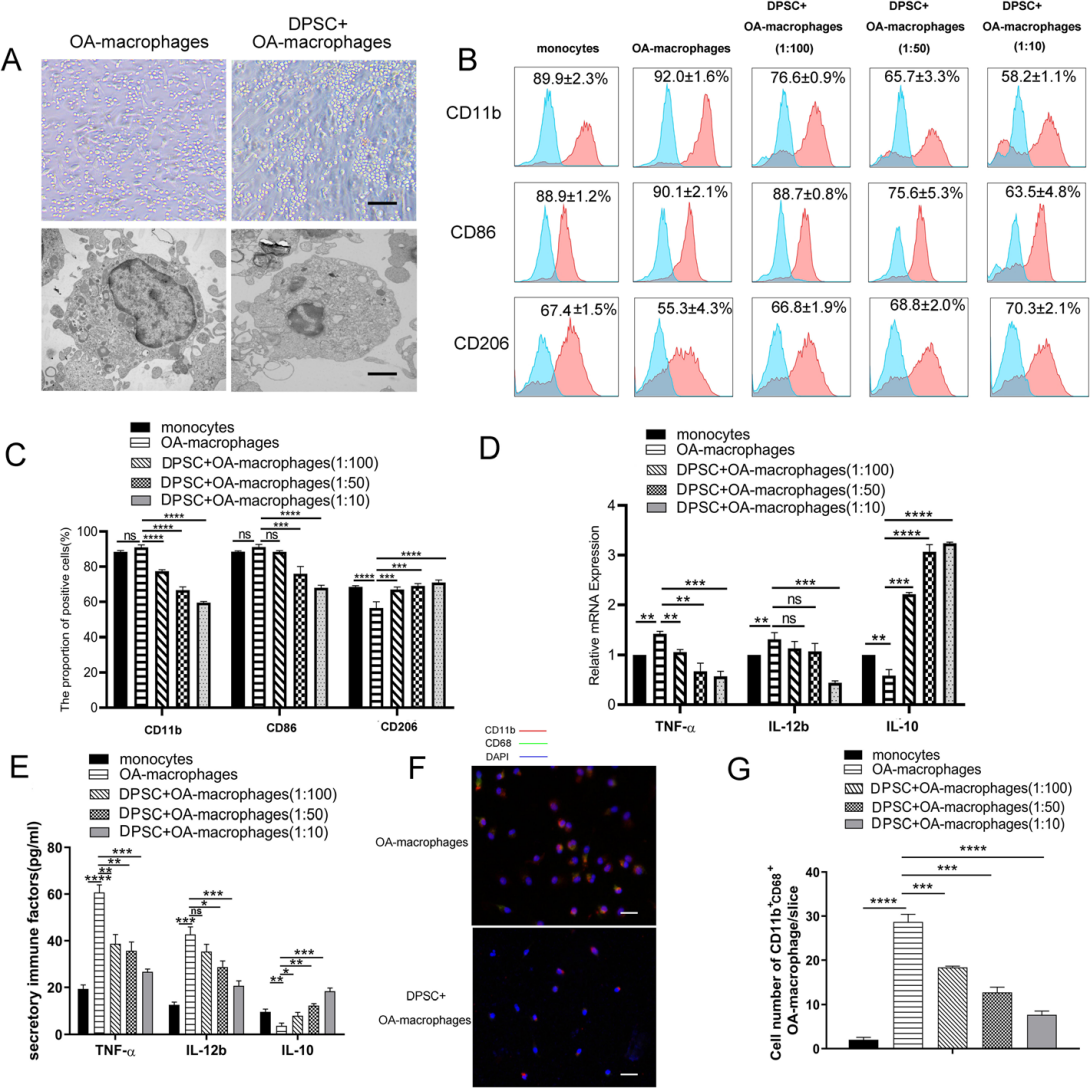
hDPSCs suppressed osteoarthritic macrophage activation in directly coculture system in vitro. hDPSCs suppressed the pseudopodia formation and reduced lysosomes in osteoarthritic macrophages (**a**). In addition, hDPSC-educated osteoarthritic macrophages exhibited decreased CD11b and CD86 but increased CD206 expression in a hDPSC dose-dependent way (**b**, **c**). Blue curve represents unstained cells used as control. Furthermore, hDPSCs significantly suppressed the mRNA expression and protein secretion of IL-12b and TNF-α and increased the expression of IL-10 in osteoarthritic macrophages (mRNA expression in **d** and protein secretion in **e**). Moreover, hDPSCs downregulated the expression of inflammatory molecule CD11b^+^CD68^+^ in osteoarthritic macrophages in vitro (**f** and **g**), indicated the hDPSCs decreased the number of CD11b^+^CD68^+^ osteoarthritic macrophages. **P < 0.01, ***P < 0.001; ****P < 0.0001. Bars in **a** upper row, **a** lower row, and **f** represent 200μm, 20μm, and 200μm, respectively. hDPSCs, human dental pulp stem cells; IL-12b, interleukin 12; TNF-α, tumor necrosis factor α; IL-10, interleukin 10

**Fig. 2:**
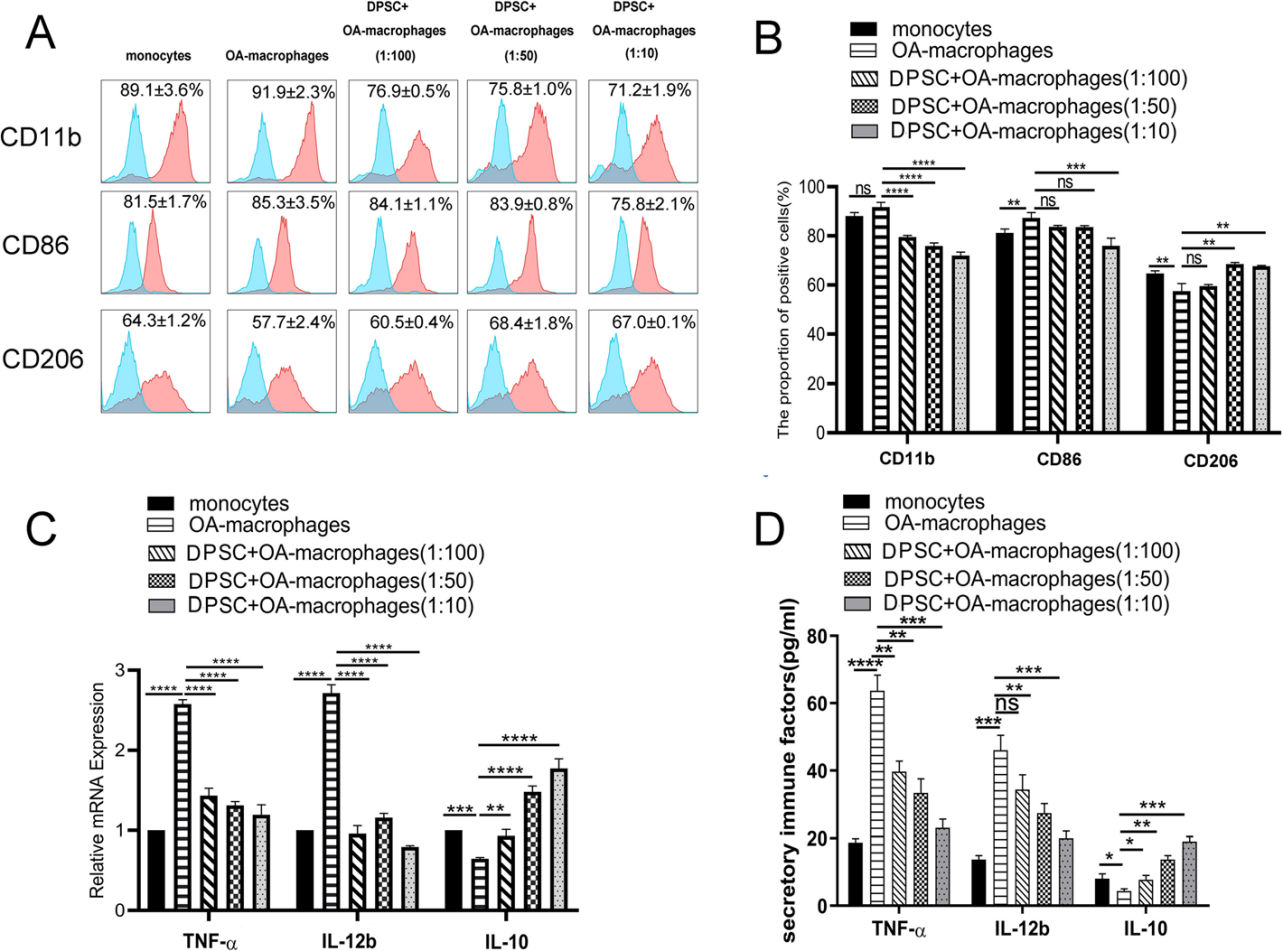
hDPSCs suppress osteoarthritic macrophages without intercellular contact. In the current study, a Transwell chamber system was used to separate hDPSCs (lower compartment) from macrophages (upper compartment). hDPSCs markedly suppressed the expression of stimulatory molecules, including CD11b and CD86 in the Transwell chamber system (**a**, **b**). In addition, hDPSCs significantly prevented the mRNA and secretion level of macrophage derived immune factors, including TNF-α and IL-12b, and promoted IL-10 expression without direct cell-cell contact (mRNA expression in **c** and protein secretion in **d**), *P < 0.05, **P < 0.01, ***P < 0.001; ****P < 0.0001. hDPSCs, human dental pulp stem cells; IL-12b, interleukin 12; TNF-α, tumor necrosis factor α; IL-10, interleukin 10

**Fig. 3:**
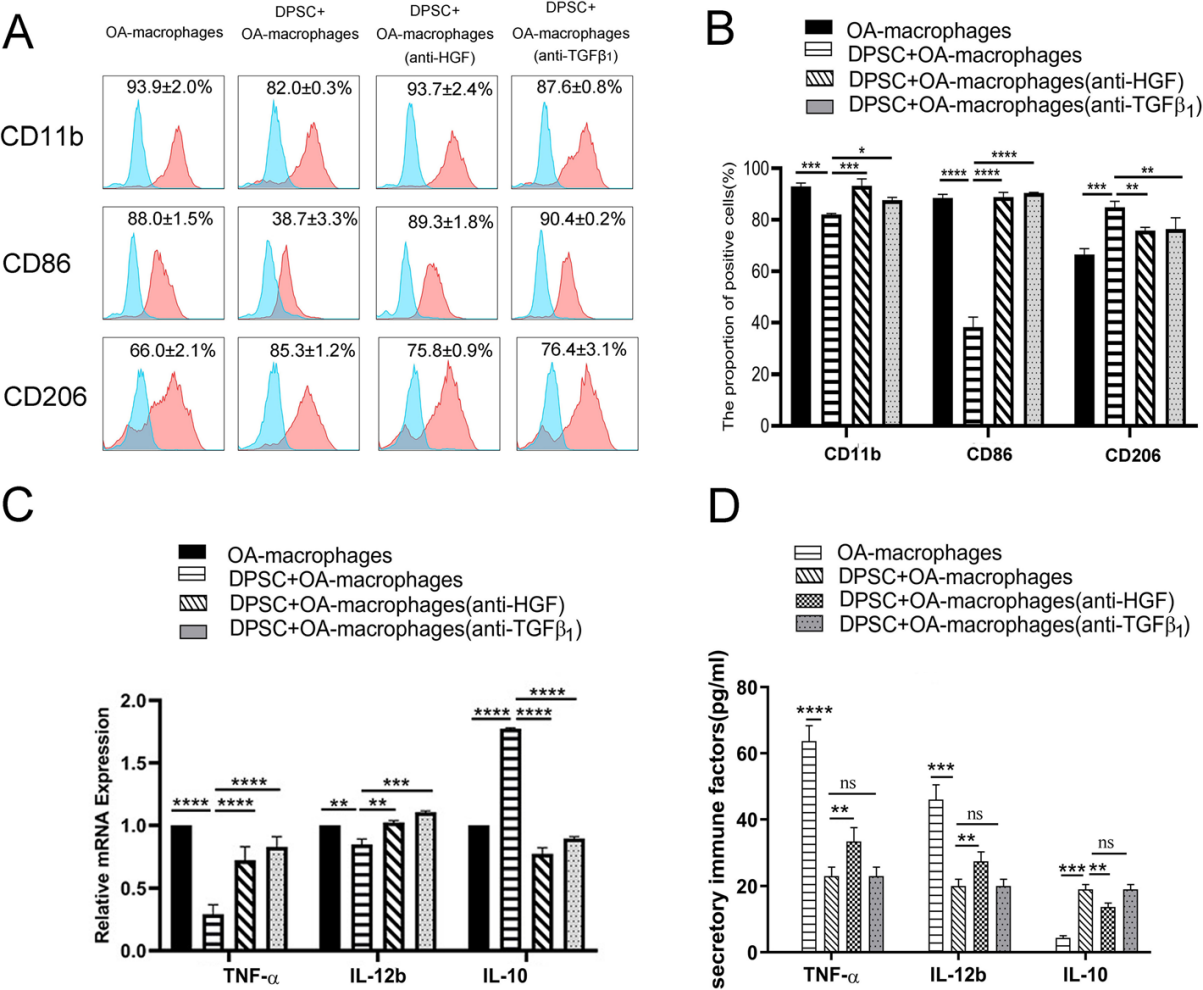
hDPSCs suppress osteoarthritic macrophages partially by secreting HGF and TGF-β1. In the current study, the expression levels of CD11b and CD86 were significantly reverted but the expression level of CD206 were remarkably abolished by direct supplementation with anti-TGF-β1 (200 ng/ml) or anti-HGF (200 ng/ml) neutralizing antibodies in the presence of hDPSCs (hDPSCs to osteoarthritic macrophage, 1:10) (**a**, **b**). In addition, the mRNA and secretion level of TNF-α and IL-12b increased and that of IL-10 decreased (mRNA expression in **c** and protein secretion in **d**), *P < 0.05, **P < 0.01,***P < 0.001; ****P < 0.0001. hDPSCs, human dental pulp stem cells; TGF-β1, transforming grow factor beta 1; HGF, hepatocyte growth factor; IL-12b, interleukin 12; TNF-α, tumor necrosis factor α; IL-10, interleukin 10

**Fig. 4:**
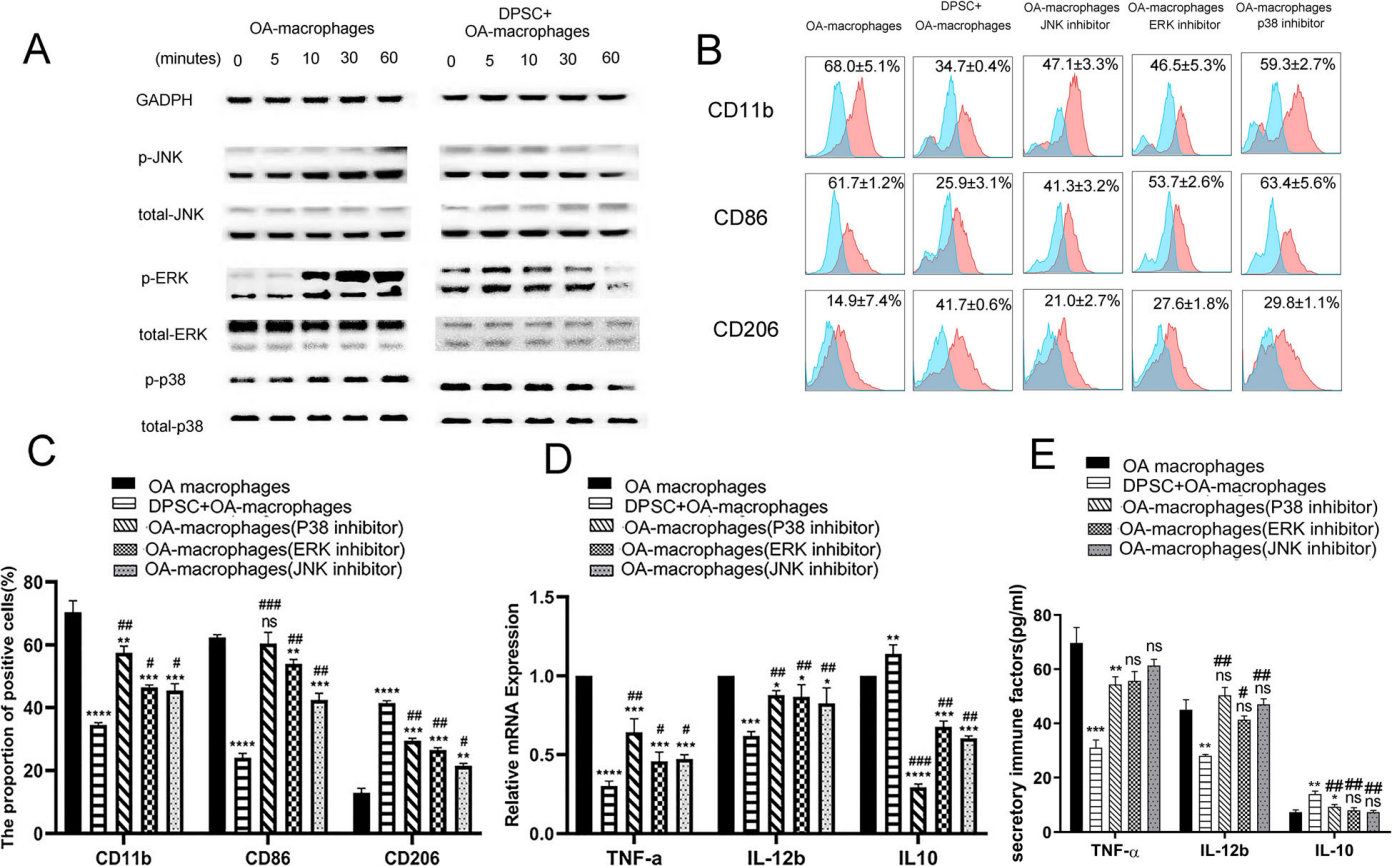
hDPSCs suppress osteoarthritic macrophages partially by inactivating MAPK pathways. The JNK/SAPK, ERK/MAPK, and p38/MAPK pathways in macrophages were markedly activated by OASF in a time-dependent manner while exposure of osteoarthritic macrophages to hDPSCs conditional medium suppressed the activation of these pathways in macrophages (**a**). Additionally, specific chemical pathway inhibitors, including SB203580 (for p38/MAPK), PD98059 (for ERK/MAPK), and JNK inhibitor II (for JNK/SAPK) showed a similar effect on the immunophenotype, and expression of TNF-α, IL-12b, and IL-10 of osteoarthritic macrophages to that of hDPSCs. Notably, blockage of single pathway could not result in same suppression on osteoarthritic macrophage as that of hDPSCs (immunophenotypes in **b** and **c**, mRNA expression in **d** and protein secretion in **e**), *P < 0.05, **P < 0.01, ***P < 0.001, ****P < 0.0001, hDPSC group and inhibitor groups compared to osteoarthritic macrophage group) (**b**–**e**, ^#^*P* < 0.05, ^##^*P* < 0.01, ^###^*P* < 0.001, inhibitor groups compared to hDPSC group). hDPSCs, human dental pulp stem cells; IL-12b, interleukin 12; TNF-α, tumor necrosis factor α; IL-10, interleukin 10

**Fig. 5:**
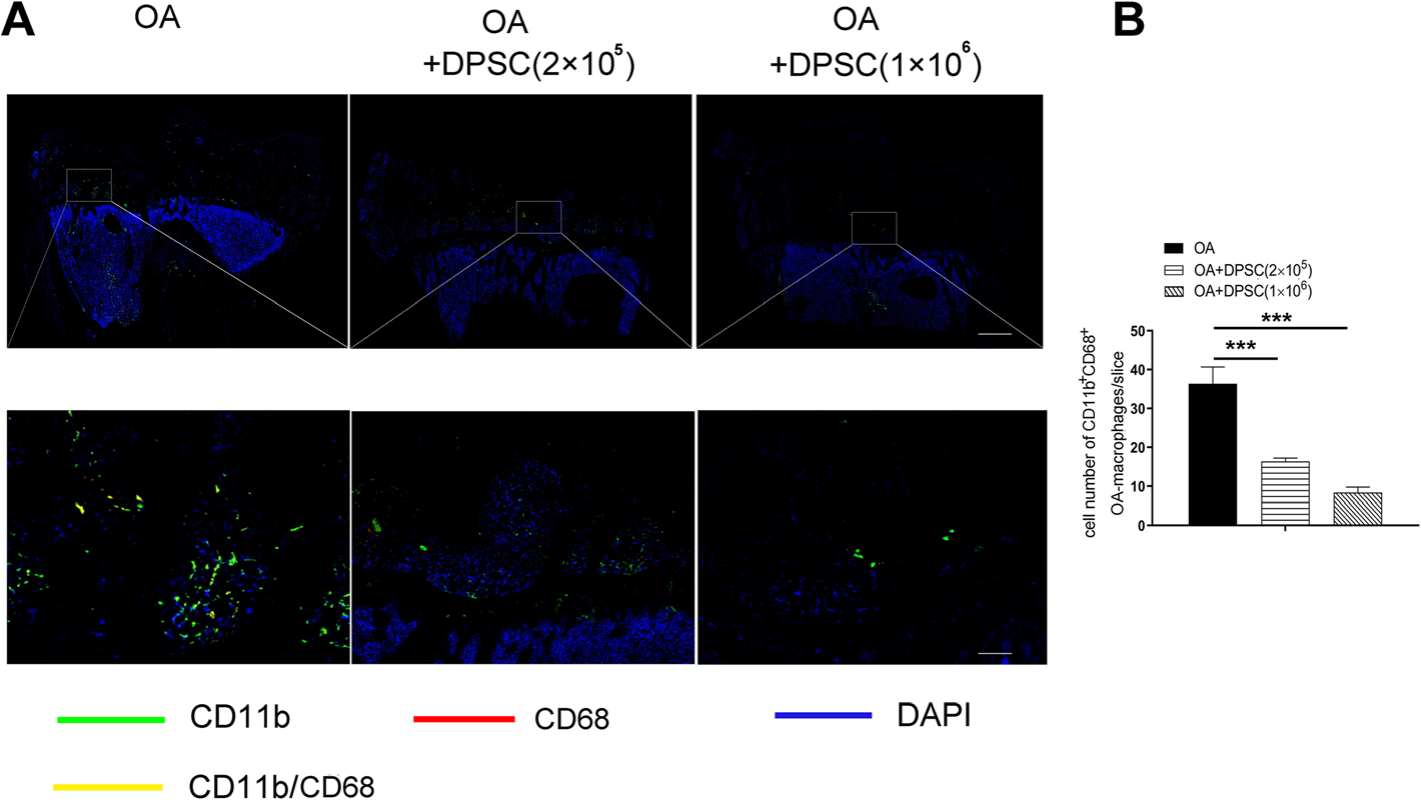
hDPSCs suppress osteoarthritic macrophage activation in a rabbit posttraumatic knee OA model. hDPSC infusion significantly reduced CD11b^+^CD68^+^ macrophages in the host articular osteochondral tissues in an OA animal model at a hDPSC dose-dependent manner (**a**, **b**). ***P < 0.001. Bars in **a** upper row and **a** lower row represent 5mm and 200μm, respectively. hDPSCs, human dental pulp stem cells; OA, osteoarthritis

**Fig. 6:**
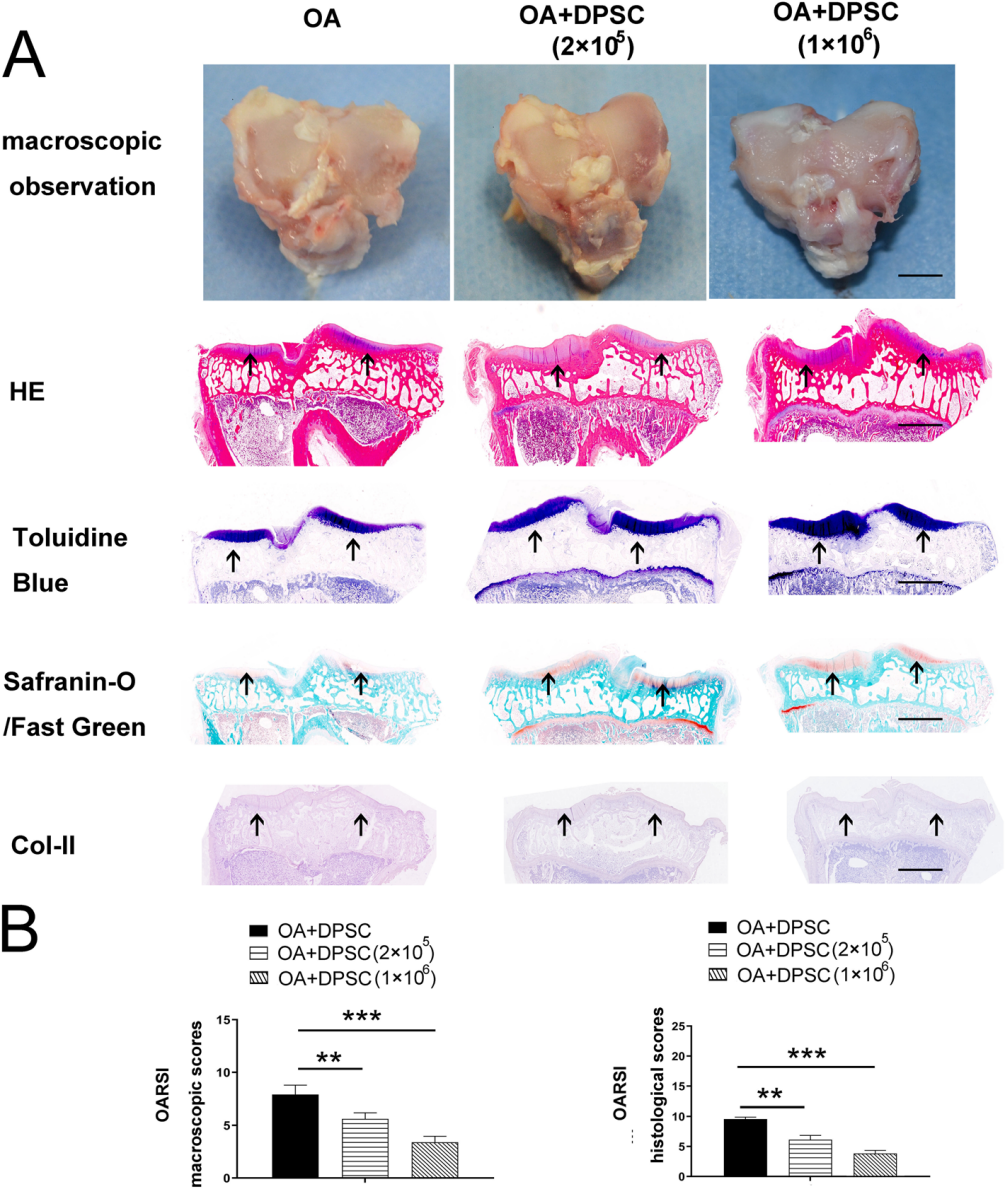
hDPSCs attenuated damage to the articular cartilage in vivo. The macroscopic characteristics of OA model group and the hDPSC treatment group are showed in **a**. In addition, the results of the HE, toluidine blue staining, safranin-O/Fast Green staining, and immunochemistry of Col-II showed that hDPSCs significantly improved the histological findings of osteochondral tissue in OA models. More intense staining in the cartilage layer was showed by using arrows, respectively (**a**). Moreover, the high-hDPSC group exhibited significantly lower pathological scores than the other 2 experimental groups (**b**). **P<0.01, ***P<0.001. Bars represent 3mm in microscopic data of **a** and 2mm in histopathological data in **a**, respectively. hDPSCs, human dental pulp stem cells; Col-II, collagen II; OA, osteoarthritis

Data are represented as the mean values with standard deviations. Statistical significance was analyzed using Student’s *t* test. The one-way ANOVA was used in multiple group data analysis. *P* values less than 0.05 were considered to be significant. In Figs. 1c–e, g and 2c–e, the Student *t*-test was used in the comparisons between “monocyte” and “OA-macrophage” groups, the one-way ANOVA was used in the four groups except “monocyte” group. In Figs. 3c–e and 4c–e and in Figure S1, the Student *t*-test was used. In Figs. 5b and 6b, the one-way ANOVA was used.

## Results

### hDPSCs strongly inhibited the activation of osteoarthritic macrophages in vitro

To investigate whether hDPSCs affected macrophage morphology, osteoarthritic macrophages, and hDPSC-treated osteoarthritic macrophages (directly co-cultured) were observed with phase contrast and transmission electron microscopy. As shown in Fig. 1a, the osteoarthritic macrophages were large cells with an irregular outline. In addition, these cells generally displayed pseudopodia, vesicles, and membrane folding on the cell surface. Moreover, the predominant cytoplasmic organelles in osteoarthritic macrophages were lysosomes, secondary lysosomes, and residual bodies. In contrast, the hDPSC-educated osteoarthritic macrophages were small and round and lacked pseudopodia. Additionally, fewer lysosomes were observed in hDPSC-educated osteoarthritic macrophages.

Cell surface molecules and immune factors are indispensable for macrophages to initiate immune responses. As showed in Fig. 1b and c, osteoarthritic macrophages expressed lower cell surface marker CD206 than that of monocytes (*p*<0.0001). Compared with osteoarthritic macrophages, hDPSC-educated osteoarthritic macrophages exhibited decreased cell surface marker CD11b at 1:100, 1:50, and 1:10 ratios (*p*<0.0001, *p*<0.0001, *p*<0.0001, respectively) and cell surface marker CD86 at 1:50 and 1:10 ratios (*p*<0.001, *p*<0.0001, respectively) but increased cell surface marker CD206 expression at 1:100, 1:50, and 1:10 ratios (*p*<0.001, *p*<0.001, *p*<0.0001, respectively) (Fig. 1b, c). Notably, the inhibitory effect was hDPSC dose-dependent.

Further investigations showed that hDPSCs suppressed the expression of key immune factors in osteoarthritic macrophages. The real-time PCR results demonstrated that osteoarthritic macrophages expressed higher mRNA level of TNF-α (*p*<0.01) and higher mRNA level of IL-12b (*p*<0.01) but lower mRNA level of IL-10 (*p*<0.01) than that of monocytes (Fig. 1d). In addition, hDPSCs significantly suppressed the mRNA expression of TNF-α at 1:100, 1:50, and 1:10 ratios (*p*<0.01, *p*<0.01, *p*<0.001, respectively) and the mRNA expression of IL-12b at 1:10 ratios (*p*<0.001) and increased the mRNA expression of IL-10 in osteoarthritic macrophages at 1:100, 1:50, and 1:10 ratios (*p*<0.001, *p*<0.001, *p*<0.0001, respectively) (Fig. 1d). Furthermore, the ELISA results demonstrated that osteoarthritic macrophages secreted higher level of TNF-α and IL-12b (*p*<0.0001, *p*<0.001, respectively) and lower level of IL-10 (*p*<0.01) than that of monocytes (Fig. 1e). Moreover, hDPSCs significantly suppressed the secretion of TNF-α by osteoarthritic macrophages at 1:100, 1:50, and 1:10 ratios (*p*<0.01, *p*<0.01, *p*<0.001, respectively) and the secretion of IL-12b at 1:50 and 1:10 ratios (*p*<0.05, *p*<0.001, respectively) and increased the secretion of IL-10 in osteoarthritic macrophages at 1:100, 1:50, and 1:10 ratios (*p*<0.05, *p*<0.01, *p*<0.001, respectively) (Fig. 1e). Moreover, immunofluorescence data demonstrated that hDPSCs downregulated the number of CD11b^+^CD68^+^ inflammatory macrophages in osteoarthritic macrophages in vitro at 1:100, 1:50, and 1:10 ratios (*p*<0.001, *p*<0.001, *p*<0.0001, respectively) (Fig. 1g, f).

### hDPSCs suppress osteoarthritic macrophages without intercellular contact

To explore whether the inhibition of osteoarthritic macrophages by hDPSCs requires intercellular contact, we used a Transwell chamber system to separate hDPSCs (lower compartment) from macrophages (upper compartment). We found that osteoarthritic macrophages expressed higher cell surface marker CD86 (*p*<0.01) but expressed lower cell surface marker CD206 than that of monocytes (*p*<0.01). In addition, hDPSCs markedly suppressed the expression of stimulatory molecules on the cell surface of osteoarthritic macrophages, including CD11b at 1:100, 1:50, and 1:10 ratios (*p*<0.0001, *p*<0.0001, *p*<0.0001, respectively) and CD86 at 1:10 ratios (*p*<0.001), but increased the expression of cell surface marker CD206 at 1:50 and 1:10 ratios (*p*<0.01, *p*<0.01, respectively) (Fig. 2a, b) in the Transwell chamber system. Notably, CD86 expression was only significantly reduced at the highest ratio of 1:10.

In addition, hDPSCs significantly prevented the mRNA level of macrophage-derived immune factors without direct cell-cell contact, including TNF-α (at 1:100, 1:50, and 1:10 ratios, *p*<0.0001, *p*<0.0001, *p*<0.0001, respectively) and IL-12b (at 1 1:50, and 1:10 ratios, *p*<0.0001, *p*<0.0001, *p*<0.0001, respectively), but promoted IL-10 mRNA expression (at 1:100, 1:50, and 1:10 ratios, *p*<0.01, *p*<0.0001, *p*<0.0001, respectively) (Fig. 2c). Moreover, ELISA results showed that in the presence of hDPSCs, osteoarthritic macrophages in Transwell chamber system secreted low level of TNF-α (at 1:100, 1:50, and 1:10 ratios, *p*<0.01, *p*<0.01, *p*<0.001, respectively) and IL-12b (at 1:50 and 1:10 ratios, *p*<0.01, *p*<0.001, respectively), and higher level of IL-10 at 1:100, 1:50, and 1:10 ratios, *p*<0.05, *p*<0.01, *p*<0.001, respectively) (Fig. 2d), which is consistent with that of mRNA data. These data indicated that soluble factors from hDPSCs were involved in the observed suppression (Fig. 2c, d). Importantly, cell contact-independent regulations on CD11b, TNF-α, and IL-10 were observed at a relatively low hDPSC/macrophage ratio (1:100). However, this was not the case for CD86 reduction and CD206 increase with flow, neither for IL-12b with ELISA, which indicated the different regulatory effects of hDPSCs on cell surface markers of OA-macrophages and inflammatory factors they secreted.

### hDPSCs suppress osteoarthritic macrophages partially by secreting HGF and TGF-β1

We further defined which factors in the supernatant were involved in the inhibitory effects by utilizing neutralizing antibodies. TGF-β1 and HGF were investigated because numerous studies have demonstrated that they are pivotal immune factors from hDPSCs and contribute to hDPSC-mediated immunoregulation. In the current study, the cell surface expression of CD11b and CD86 were significantly reverted by direct supplementation with anti-TGF-β1 (200 ng/ml) or anti-HGF (200 ng/ml) neutralizing antibodies (Fig. 3a, b, *P < 0.05, **P < 0.01, ***P < 0.001; ****P < 0.0001, respectively ) in the presence of hDPSCs (hDPSCs to osteoarthritic macrophage, 1:10). In addition, the mRNA expression of TNF-α (****P < 0.0001) and IL-12b (**P < 0.01; ****P < 0.0001, respectively) increased and that of IL-10 decreased (****P < 0.0001; ****P < 0.0001, respectively) (Fig. 3c). Moreover, the ELISA results demonstrated that the secretion of TNF-α (****P < 0.0001) and IL-12b (**P < 0.01; ****P < 0.0001, respectively) by osteoarthritic macrophages upregulated while that of IL-10 reduced (****P < 0.0001; ****P < 0.0001, respectively) (Fig. 3d). Notably, blockage of TGF-β1 exhibited limited effects on the secretion of TNF-α, IL-12b, and IL-10 by osteoarthritic macrophages (Fig. 3d).

### hDPSCs suppress osteoarthritic macrophages partially by inactivating MAPK pathways

To examine the intracellular signaling cascades, we investigated MAPK pathway protein phosphorylation in osteoarthritic macrophages because the activation of MAPK signals is critical for macrophage action [36–38]. The western-blotting results showed that the JNK/SAPK, ERK/MAPK, and p38/MAPK pathways in macrophages were markedly activated by OASF in a time-dependent manner (Fig. 4a). Most importantly, exposure of osteoarthritic macrophages to hDPSC conditional medium without any exogenous cytokine or chemical inhibitor addition caused significant inactivation of the JNK/SAPK, ERK/MAPK, and p38/MAPK pathways in macrophages (Fig. 4a and Fig. S1, *P < 0.05, **P < 0.01, ***P < 0.001).

To further clarify the association of osteoarthritic macrophages and the MAPK pathways, specific chemical pathway inhibitors, including SB203580 (for p38/MAPK), PD98059 (for ERK/MAPK), and JNK inhibitor II (for JNK/SAPK), were added to the arthritic macrophage culture system. As shown in Fig. 4b and c, hDPSCs significantly suppressed the expression of the co-stimulatory cell surface markers CD11b and CD86 but increased the expression of regulatory surface cell surface marker CD206, while JNK inhibitor II, PD98059, and SB203580 showed similar effects on osteoarthritic macrophages (hDPSCs, **P < 0.01, ***P < 0.001, ****P < 0.0001, respectively; specific inhibitors, ^#^*P* < 0.05, ^##^*P* < 0.01, ^###^*P* < 0.001, respectively). Additionally, hDPSCs decreased the mRNA level (Fig. 4d) and secretion level (Fig. 4e) of TNF-α and IL-12b and increased that of IL-10(**P < 0.01, ***P < 0.001, respectively). The changes of immune factors in the presence of the specific inhibitors were similar to that of hDPSCs group. Notably, blockage of single pathway could not result in same suppression on osteoarthritic macrophage as that of hDPSCs (Fig. 4b–e, hDPSCs, *P < 0.05, **P < 0.01, ***P < 0.001, ****P < 0.0001; respectively; specific inhibitors, ^#^*P* < 0.05, ^##^*P* < 0.01, ^###^*P* < 0.001). Thus, we suggest that OASF activated macrophages partially by activating the JNK/SAPK, ERK/MAPK, and p38/MAPK pathways, and hDPSCs might exhibit an inhibitory effect on osteoarthritic macrophages by blocking multiple pathways of MAPK signaling.

### hDPSCs suppress osteoarthritic macrophage activation in a rabbit posttraumatic knee OA model

Although in vitro studies have shown that hDPSCs are capable of suppressing osteoarthritic macrophages, it remains unknown whether this is the case in vivo. Therefore, the phenotype of osteoarthritic macrophages was determined by immunofluorescence. Promisingly, we found that hDPSC infusion significantly reduced CD11b^+^CD68^+^ macrophages in the host articular osteochondral tissues (Fig. 5a, b) (***P < 0.001). In addition, the numbers of CD11b^+^CD68^+^macrophages in the articular osteochondral tissue of hDPSC-treated rabbits were markedly decreased with the increased number of infused hDPSCs (Fig. 5a, b). The suppressive effects of hDPSCs on osteoarthritic macrophages in an OA animal model in vivo are consistent with those observed in an in vitro cell model.

### hDPSCs attenuated damage to the articular cartilage in vivo

At 10 weeks after hDPSCs’ injection, the representative macroscopic observation displayed discriminatory regenerated tissue between the OA model group and the hDPSC treatment group. The articular surfaces in the untreated group were not as smooth as those in the hDPSC-treated group (Fig. 6a). Large osteophytes were observed at the periphery of the tibial plate. In contrast, no obvious osteochondral lesions in articular cartilage were observed in the hDPSC-treated groups (Fig. 6a). The histological evidence further supported the gross morphological findings. As shown in Fig. 6a, HE staining showed that hDPSC administration alleviated tissue erosion in OA keen joints. The toluidine blue, safranin-O/Fast Green staining, and immunochemistry of Col-II showed that hDPSCs significantly improved the histological findings associated with structure and proteoglycans in a cell dose-dependent manner (Fig. 6a). Quantitatively, the high-hDPSC group exhibited significantly lower pathological scores than the other 2 experimental groups (Fig. 6b, **P<0.01, ***P<0.001).

## Discussion

In the current study, we demonstrated the suppressive effects of clinical-grade hDPSCs on the activation of osteoarthritic macrophages in vitro. Additionally, we found that hDPSC injection significantly reduced the activation of osteoarthritic macrophages in vivo and alleviated cartilaginous damage in knee OA.

The preparation of hDPSCs is less invasive and more feasible and involves fewer ethical issues than their counterparts in other tissues. In addition, accumulated evidence has demonstrated that hDPSCs share similar characteristics with bone marrow MSCs, which potentiates their application in treating intractable diseases, including Alzheimer’s disease, cardiac ischemia-reperfusion injury, and diabetes [16, 39, 40]. Moreover, hDPSCs have exhibited promising potential in the treatment of oral and skeletal disorders due to their high osteochondral capacity and immunoregulatory properties. Asutay et al. reported that hDPSCs mixed with hydroxyaptite-tricalcium phosphatel paste yield a high calcification rate and bone mineral density value in a rat calvarial defect model [41]. Further radiographic data showed that bone regeneration in the presence of DPSC-hydroxyapatite-tricalcium phosphatel was greater than that without DPSC, which suggests that DPSCs may be a suitable factor for skeletal tissue engineering [41]. Our previous study demonstrated that transplantation of hDPSCs markedly prevented bone loss in the early phase of ovariectomy-induced osteoporosis [28].

Macrophages are hematopoietic stem cell-derived immune cells that have a pivotal role in innate and adaptive immune responses. Activated macrophages are capable of taking up innate or antigens or invading pathogens and highly express costimulatory molecules and secrete more immune cytokines, which are indispensable for the immune response and tissue regeneration [10]. However, recent studies have revealed that macrophage activation contributes to the pathological progression of OA. Xie et al. reported that activated macrophages enhance the progression of OA by increasing the secretion of matrix metalloproteinases [10]. Moreover, macrophages in the subchondral bone are not only involved in chondro-osteogenic remodeling but also cause joint pain by releasing inflammatory factors [42, 43]. Thus, a full understanding of the regulation of osteoarthritic macrophages will definitely shed light on OA therapy.

Macrophage activation is greatly dependent upon stromal microenvironments [44, 45]. Serbulea et al. reported that the adipose tissue microenvironment controls the macrophage phenotype either to maintain lean tissue homeostasis or to drive inflammation [45]. Li et al. found that the stromal niche of gastric cancer contributed to macrophage polarization within the gastric cancer niche through considerable secretion of IL-6 and IL-8 [46]. Additionally, our previous studies demonstrated that MSCs were capable of controlling the differentiation fate of monocytes, which are important progenitors of macrophages [20, 23]. Thus, we explored the possibility of osteoarthritic macrophage control by hDPSCs. In the current study, we found that hDPSCs could suppress CD11b, CD86, and CD68 expression but increase CD206 expression. Functionally, we found that hDPSCs inhibited the expression of IL-12b and TNF-α and promoted the production of IL-10 in the present study. This observation indicates that hDPSCs suppressed the activation of osteoarthritic macrophages and further investigations are need to explore whether osteoarthritic macrophages develop into a new type of macrophage [12, 43].

Previous reports have shown that direct cell-cell interactions of macrophages with neighboring stromal cells are required for MSC-mediated suppression [47–49]. However, in the current study, the suppressive effects of hDPSCs on osteoarthritic macrophages generation in the Transwell chamber system remained, which suggested that hDPSCs suppress osteoarthritic macrophages mainly by secreting soluble factors. Numerous reports have demonstrated that hDPSCs highly express HGF, and we previously found that HGF has a pivotal role in controlling the regenerative properties of hDPSCs [27]. hDPSCs exerted multifaceted benefits for treating experimental rheumatoid arthritis by secreting factors [42]. In the early phase of rheumatoid arthritis progression, HGF overexpression in hDPSCs inhibited pathological aggression by its immunosuppressive effects [27]. In the late phase of rheumatoid arthritis, HGF promoted synovitis by activating fibroblast-like synoviocytes to produce pathogenic IL-6, which accelerated cell proliferation and induced apoptosis resistance. Thus, in the current study, we blocked hDPSC-derived HGF, and the suppressive effects on osteoarthritic macrophages were partially reverted, which revealed the novel role of HGF in the hDPSCs-mediated regenerative capacity. In addition to HGF, we found that TGF-β1 contributes to the inhibition of osteoarthritic macrophages by hDPSCs by using neutralization antibody. The findings are consistent with previous reports that TGF-β1 are closely involved in MSCs mediated immunoregulation. Cen et al. reported that human bone marrow MSCs promoted CD4 ^+^ T cell migration and differentiation through C-X-C motif chemokine ligand 8 and TGF-β1, and autophagy of MSCs enhanced the promotion effects on T cells [50]. Our previous work showed that suspending MSCs derived culture medium suppressed Concanavalin A induced T cell proliferation [51]. In addition, suspending MSCs expressed transforming factor β1 and interleukin-6 at a higher level compared to that of adherent MSCs [51]. Collectively, these data suggested that soluble factors, including HGF and TGF-β1, contribute to hDPSC-mediated suppression of OA macrophages, but we are aware that other unknown secreted factors may have a potential role in the suppressive effects. In addition, it is unclear if hDPSC treatment can increase HGF or TGF-β1 levels in vivo systemically or locally, which is important to link in vitro studies with in vivo model treatment and need further explorations in future studies.

To understand the molecular mechanism underlying the suppressive effect of hDPSCs on osteoarthritic macrophages, the MAPK pathway was chosen for investigation, as it was reported to be involved in macrophage activation in inflammatory microenvironments [26, 36–39]. OASF induced marked activation of the p38/MAPK, ERK/MAPK, and JNK/SAPK pathways in osteoarthritic macrophages. Notably, inhibition of these pathways with specific chemical inhibitors changed the expression of co-stimulatory molecules and the production of inflammatory factors in macrophages, indicating that the p38/MAPK, ERK/MAPK, and JNK/SAPK pathways are involved in osteoarthritic macrophage activation. In the current study, hDPSC conditional medium significantly suppressed MAPK signal transduction in activated macrophages. However, a single pathway blockade could not completely suppress the activation of macrophages, indicating that some other mechanisms are also involved in OASF stimulation. In addition, it needs further investigations to explore whether HGF and TGF-β1 activate MAPK signaling pathways in OA macrophages in future studies. Moreover, if HGF/TGF-β1 recombinant proteins or MAPK inhibitors be used as future therapeutic targets for OA patients in clinic need more preclinical studies, especially data of bigger OA animal models and underlying mechanisms.

Notably, our data demonstrated that hDPSCs suppressed macrophage activation in vivo. Most importantly, the hDPSCs were capable of alleviating cartilaginous damage in OA knee joints, which implies the potential application of hDPSCs in the therapy of OA. Our findings showed marked infiltration of inflammatory macrophages (CD11b^+^CD68^+^) in the subchondral bones and articular cartilage of OA rabbits, which have been reported to be involved in the osteochondral remodeling of OA joints [12, 43, 52–55]. Promisingly, hDPSC infusion significantly reduced the number of inflammatory macrophages (CD11b^+^CD68^+^) in articular osteochondral tissue of OA rabbits. The change in the macrophages may contribute to the protective effects of hDPSCs in OA. Consistent with the macrophage alteration, further pathological analysis validated that hDPSCs contribute to the settlement of OA. The HE, toluidine blue staining, safranin-O/Fast Green, and immunochemistry of Col-II staining showed fewer lesions of the knee articular tissue post hDPSC infusion. Moreover, the suppressive effect of hDPSCs on OA macrophages was strengthened with the increase of the cell number of hDPSCs, which indicated that different dosages of hDPSCs may yield different therapeutic effects. However, we are aware that other mechanisms may contribute to the regenerative effects of hDPSCs.

Nevertheless, we must acknowledge several limitations in our study. First, our data are based on only one kind of OA animal model; studies from more OA models are required to further validate the findings. Second, to determine the cellular and molecular mechanisms that contribute to the therapeutic effects of hDPSCs on OA, high-throughput assays may be helpful. Third, the time point of hDPSC injection may influence the outcome of OA. Further investigations of the relevance of MSC treatment time and therapeutic results should be performed in the future. Fourth, we are aware that osteoarthritic macrophages were generated from peripheral blood monocytes in vitro induction by exposure OASF. There may be differences regarding macrophages naturally resident in OA joints and further analysis on the in vivo osteoarthritic macrophages is needed in future study. Fifth, though we have reported the changes of surface markers and expression/secretion that contribute to the activation of osteoarthritic macrophages in the current study, there may be other unknown changes of the hDPSC-treated osteoarthritic macrophages, and a high-through assay will be helpful to reveal it in future study. Sixth, the tissue assessment should be performed at more time-points or more samples such as mediator secretion in synovial fluid should be included to further valid the findings in the current study.

## Conclusion

In the present study, we reported the effect of hDPSCs on osteoarthritic macrophages and the effect of hDPSC injection on a rabbit OA model. Our study shows the suppressive effects of hDPSCs on osteoarthritic macrophages in vitro and in vivo and the protective role of hDPSCs on cartilaginous tissues in the OA knee joint, which suggests that hDPSCs may be potential candidates for stem cell-based OA therapy. However, the effects of hDPSCs seemed to be mostly mediated by paracrine mechanisms, including, but not limited to, TGF-β1 and HGF, and acting at the level of MAPK signaling pathways. Thus, a comprehensive analysis of therapeutic effects in more animal models and high-throughput assays is needed to further clarify the underlying mechanisms.

## Supplementary Information

**Additional file 1: Table S1.**

**Additional file 2: Table S2–S4.**

**Additional file 3: Table S5.**

**Additional file 4: Figure S1.** The MAPK pathways are closely involved in OA macrophage activation. The OASF activated MAPK pathway in OA macrophages in a time dependent manner. The conditional medium from hDPSCs significantly suppressed the activation in OA macrophages induced by OASF. The data are generated from western-blotting data from Fig. 4a by using greyscale software Fiji. *, *P*<0.05, **, *P*<0.01, ***, *P*<0.001. OASF: Synovial fluid from OA patients; OA: osteoarthritis.

## Data Availability

The datasets used and/or analyzed during the current study are available from the corresponding author on reasonable request.

## Abbreviations

hDPSCs: Human dental pulp stem cells
OA: Osteoarthritis
IL-12: Interleukin 12
TNF-α: Tumor necrosis factor α
OASF: Synovial fluid from OA patients
MSCs: Mesenchymal stem cells
HGF: Hepatocyte growth factor
TGF-β1: Transforming growth factor beta 1
PCR: Polymerase chain reaction
M-CSF: Microphage colony stimulating factor
IFN-γ: Interferon γ
FBS: Fetal bovine serum
FTIC: Fluorescein isothiocyanate
PE: Phycoerythrin
APC: Allophycocyanin

## References

[1] Findlay DM, Kuliwaba SK (2016). Bone-cartilage crosstalk: a conversation for understanding osteoarthritis. Bone Res..

[2] Coryell PR, Diekman BO, Loeser RF (2021). Mechanisms and therapeutic implications of cellular senescence in osteoarthritis. Nat Rev Rheumatol..

[3] Mazzei DR, Ademola A, Abbott JH (2020). Are education, exercise and diet interventions a cost-effective treatment to manage hip and knee osteoarthritis? A systematic review. Osteoarthritis Cartilage.

[4] Bannuru RR, Osani MC, Vaysbrot EE, Arden NK, Bennell K, Bierma-Zeinstra SMA, Kraus VB, Lohmander LS, Abbott JH, Bhandari M, Blanco FJ, Espinosa R, Haugen IK, Lin J, Mandl LA, Moilanen E, Nakamura N, Snyder-Mackler L, Trojian T, Underwood M, McAlindon TE (2019). OARSI guidelines for the non-surgical management of knee, hip, and polyarticular osteoarthritis. Osteoarthritis Cartilage..

[5] van den Bosch MHJ (2020). Osteoarthritis year in review 2020: biology. Osteoarthritis Cartilage.

[6] Daghestani HN, Pieper CF, Kraus VB (2015). Soluble macrophage biomarkers indicate inflammatory phenotypes in patients with knee osteoarthritis. Arthritis Rheumatol..

[7] Hsueh MF, Zhang X, Wellman SS, et al. Synergistic roles of macrophages and neutrophils in osteoarthritis progression. Arthritis Rheumatol. 2020. 10.1002/art.41486 Online ahead of print. 10.1002/art.41486 PMC7876152 32783329

[8] Smiljanovic B, Radzikowska A, Kuca-Warnawin E, Kurowska W, Grün JR, Stuhlmüller B, Bonin M, Schulte-Wrede U, Sörensen T, Kyogoku C, Bruns A, Hermann S, Ohrndorf S, Aupperle K, Backhaus M, Burmester GR, Radbruch A, Grützkau A, Maslinski W, Häupl T (2018). Monocyte alterations in rheumatoid arthritis are dominated by preterm release from bone marrow and prominent triggering in the joint. Ann Rheum Dis..

[9] Zhang H, Cai D, Bai X (2020). Macrophages regulate the progression of osteoarthritis. Osteoarthritis Cartilage..

[10] Xie J, Huang Z, Yu X, Zhou L, Pei F (2019). Clinical implications of macrophage dysfunction in the development of osteoarthritis of the knee. Cytokine Growth Factor Rev..

[11] Haraden CA, Huebner JL, Hsueh MF, Li YJ, Kraus VB (2019). Synovial fluid biomarkers associated with osteoarthritis severity reflect macrophage and neutrophil related inflammation. Arthritis Res Ther..

[12] Manferdini C, Paolella F, Gabusi E, Gambari L, Piacentini A, Filardo G, Fleury-Cappellesso S, Barbero A, Murphy M, Lisignoli G (2017). Adipose stromal cells mediated switching of the pro-inflammatory profile of M1-like macrophages is facilitated by PGE2: in vitro evaluation. Osteoarthritis Cartilage..

[13] Fahy N, de Vries-van Melle ML, Lehmann J (2014). Human osteoarthritic synovium impacts chondrogenic differentiation of mesenchymal stem cells via macrophage polarisation state. Osteoarthritis Cartilage.

[14] van der Kraan PM (2019). The interaction between joint inflammation and cartilage repair. Tissue Eng Regen Med..

[15] Li Z, Jiang CM, An S, Cheng Q, Huang YF, Wang YT, Gou YC, Xiao L, Yu WJ, Wang J (2014). Immunomodulatory properties of dental tissue-derived mesenchymal stem cells. Oral Dis..

[16] Shi X, Mao J, Liu Y (2020). Pulp stem cells derived from human permanent and deciduous teeth: Biological characteristics and therapeutic applications. Stem Cells Transl Med..

[17] Yamada Y, Nakamura-Yamada S, Umemura-Kubota E, Baba S (2019). Diagnostic cytokines and comparative analysis secreted from exfoliated deciduous teeth, dental pulp, and bone marrow derived mesenchymal stem cells for functional cell-based therapy. Int J Mol Sci..

[18] Lee S, Zhang QZ, Karabucak B, Le AD (2016). DPSCs from inflamed pulp modulate macrophage function via the TNF-α/IDO axis. J Dent Res..

[19] Ding L, Han DM, Zheng XL, Yan HM, Xue M, Liu J, Zhu L, Li S, Mao N, Guo ZK, Ning HM, Wang HX, Zhu H (2021). A study of human leukocyte antigen-haploidentical hematopoietic stem cells transplantation combined with allogenic mesenchymal stem cell infusion for treatment of severe aplastic anemia in pediatric and adolescent patients. Stem Cells Transl Med..

[20] Li X, Ding L, Wang YX, Li ZL, Wang Q, Zhao ZD, Zhao S, Wang H, Wu CT, Mao N, Zhu H (2020). Skeletal stem cell-mediated suppression on inflammatory osteoclastogenesis occurs via concerted action of cell adhesion molecules and osteoprotegerin. Stem Cells Transl Med..

[21] Wang K, Li J, Li Z, Wang B, Qin Y, Zhang N, Zhang H, Su X, Wang Y, Zhu H (2019). Chondrogenic progenitor cells exhibit superiority over mesenchymal stem cells and chondrocytes in platelet-rich plasma scaffold-based cartilage regeneration. Am J Sports Med..

[22] Zhang H, Li ZL, Yang F, Zhang Q, Su XZ, Li J, Zhang N, Liu CH, Mao N, Zhu H (2018). Radial shockwave treatment promotes human mesenchymal stem cell self-renewal and enhances cartilage healing. Stem Cell Res Ther..

[23] Zhu H, Yang F, Tang B, Li XM, Chu YN, Liu YL, Wang SG, Wu DC, Zhang Y (2015). Mesenchymal stem cells attenuated PLGA-induced inflammatory responses by inhibiting host DC maturation and function. Biomaterials..

[24] Zhu H, Guo ZK, Jiang XX, Li H, Wang XY, Yao HY, Zhang Y, Mao N (2010). A protocol for isolation and culture of mesenchymal stem cells from mouse compact bone. Nat Protoc..

[25] He J, Yan J, Wang J, et al. Dissecting human embryonic skeletal stem cell ontogeny by single-cell transcriptomic and functional analyses. Cell Res. 2021. 10.1038/s41422-021-00467-z Online ahead of print. 10.1038/s41422-021-00467-z PMC8249634 33473154

[26] Liang JW, Li PL, Wang Q, et al. Ferulic acid promotes bone defect repair after radiation by maintaining the stemness of skeletal stem cells. Stem Cells Transl Med. 2021. accepted. 10.1002/sctm.20-0536. 10.1002/sctm.20-0536 PMC8284777 33750031

[27] Dong X, Kong F, Liu C, Dai S, Zhang Y, Xiao F, Zhang H, Wu CT, Wang H (2020). Pulp stem cells with hepatocyte growth factor overexpression exhibit dual effects in rheumatoid arthritis. Stem Cell Res Ther..

[28] Kong F, Shi X, Xiao F, Yang Y, Zhang X, Wang LS, Wu CT, Wang H (2018). Transplantation of hepatocyte growth factor-modified dental pulp stem cells prevents bone loss in the early phase of ovariectomy-induced osteoporosis. Hum Gene Ther..

[29] Meng H, Hu L, Zhou Y, Ge Z, Wang H, Wu CT, Jin J (2020). A sandwich structure of human dental pulp stem cell sheet, treated dentin matrix, and matrigel for tooth root regeneration. Stem Cells Dev..

[30] Ye Q, Wang H, Xia X, Zhou C, Liu Z, Xia ZE, Zhang Z, Zhao Y, Yehenala J, Wang S, Zhou G, Hu K, Wu B, Wu CT, Wang S, He Y (2020). Safety and efficacy assessment of allogeneic human dental pulp stem cells to treat patients with severe COVID-19: structured summary of a study protocol for a randomized controlled trial (Phase I / II). Trials..

[31] Rebai MA, Sahnoun N, Abdelhedi O, Keskes K, Charfi S, Slimi F, Frikha R, Keskes H (2020). Animal models of osteoarthritis: characterization of a model induced by Mono-Iodo-Acetate injected in rabbits. Libyan J Med..

[32] Laverty S, Girard CA, Williams JM, Hunziker EB, Pritzker KPH (2010). The OARSI histopathology initiative - recommendations for histological assessments of osteoarthritis in the rabbit. Osteoarthritis Cartilage..

[33] Steigedal TS, Prestvik SW, Selvik LM (2013). Gastrin-induced proliferation involves MEK partner 1 (MP1). In Vitro Cell Dev Biol Anim..

[34] Yeo SG, Won YS (2013). LeeHY, et al. Increased expression of pattern recognition receptors and nitric oxide synthase in patients with endometriosis. Int J Med Sci..

[35] Sun Q, Li F, Li H, Chen RH, Gu YZ, Chen Y, Liang HS, You XR, Ding SS, Gao L, Wang YL, Qin MD, Zhang XG (2015). Amniotic fluid stem cells provide considerable advantages in epidermal regeneration: B7H4 creates a moderate inflammation microenvironment to promote wound repair. Sci Rep..

[36] Sun Y, Zuo Z, Kuang Y (2020). An emerging target in the battle against osteoarthritis: macrophage polarization. Int J Mol Sci..

[37] Yang G, Fan M, Zhu J, Ling C, Wu L, Zhang X, Zhang M, Li J, Yao Q, Gu Z, Cai X (2020). A multifunctional anti-inflammatory drug that can specifically target activated macrophages, massively deplete intracellular H(2)O(2), and produce large amounts CO for a highly efficient treatment of osteoarthritis. Biomaterials..

[38] Kim JH, Studer RK, Vo NV, Sowa GA, Kang JD (2009). p38 MAPK inhibition selectively mitigates inflammatory mediators and VEGF production in AF cells co-cultured with activated macrophage-like THP-1 cells. Osteoarthritis Cartilage..

[39] Mita T, Furukawa-Hibi Y, Takeuchi H, Hattori H, Yamada K, Hibi H, Ueda M, Yamamoto A (2015). Conditioned medium from the stem cells of human dental pulp improves cognitive function in a mouse model of Alzheimer’s disease. Behav Brain Res..

[40] Datta I, Bhadri N, Shahani P, Majumdar D, Sowmithra S, Razdan R, Bhonde R (2017). Functional recovery upon human dental pulp stem cell transplantation in a diabetic neuropathy rat model. Cytotherapy..

[41] Asutay F, Polat S, Gül M, Subaşı C, Kahraman SA, Karaöz E (2015). The effects of dental pulp stem cells on bone regeneration in rat calvarial defect model: micro-computed tomography and histomorphometric analysis. Arch Oral Biol..

[42] Ishikawa J, Takahashi N, Matsumoto T, Yoshioka Y, Yamamoto N, Nishikawa M, Hibi H, Ishigro N, Ueda M, Furukawa K, Yamamoto A (2016). Factors secreted from dental pulp stem cells show multifaceted benefits for treating experimental rheumatoid arthritis. Bone..

[43] Aso K, Shahtaheri SM, Hill R, Wilson D, McWilliams DF, Walsh DA (2019). Associations of symptomatic knee osteoarthritis with histopathologic features in subchondral bone. Arthritis Rheumatol..

[44] Stoppiello LA, Mapp PI, Wilson D, Hill R, Scammell BE, Walsh DA (2014). Structural associations of symptomatic knee osteoarthritis. Arthritis Rheumatol..

[45] Serbulea V, Upchurch CM, Schappe MS, Voigt P, DeWeese DE, Desai BN, Meher AK, Leitinger N (2018). Macrophage phenotype and bioenergetics are controlled by oxidized phospholipids identified in lean and obese adipose tissue. Proc Natl Acad Sci U S A..

[46] Li W, Zhang X, Wu F, Zhou Y, Bao Z, Li H, Zheng P, Zhao S (2019). Gastric cancer-derived mesenchymal stromal cells trigger M2 macrophage polarization that promotes metastasis and EMT in gastric cancer. Cell Death Dis..

[47] Pajarinen J, Lin T, Gibon E, Kohno Y, Maruyama M, Nathan K, Lu L, Yao Z, Goodman SB (2019). Mesenchymal stem cell-macrophage crosstalk and bone healing. Biomaterials..

[48] Özdemir AT, Özgül Özdemir RB, Kırmaz C, Sarıboyacı AE, Ünal Halbutoğlları ZS, Özel C, Karaöz E (2016). The paracrine immunomodulatory interactions between the human dental pulp derived mesenchymal stem cells and CD4 T cell subsets. Cell Immunol..

[49] Wang D, Zhu NX, Qin M, Wang YY (2019). Betamethasone suppresses the inflammatory response in LPS-stimulated dental pulp cells through inhibition of NF-kappaB. Arch Oral Biol..

[50] Cen SZ, Wang P, Xie ZY (2019). Autophagy enhances mesenchymal stem cell-mediated CD4 + T cell migration and differentiation through CXCL8 and TGF-β1. Stem Cell Res Ther.

[51] Li X, Wu WQ, Ding L, Liu YL, Mao N, Zhang Y, Zhu H, Ning SB (2016). Modulatory effect of mouse compact bone-derived suspending MSC on T cells and it’s related mechanisms. Zhongguo Shi Yan Xue Ye Xue Za Zhi..

[52] Zhou F, Mei J, Han X, Li H, Yang S, Wang M, Chu L, Qiao H, Tang T (2019). Kinsenoside attenuates osteoarthritis by repolarizing macrophages through inactivating NF-kappaB/MAPK signaling and protecting chondrocytes. Acta Pharm Sin B..

[53] de Visser HM, Korthagen NM, Müller C, Ramakers RM, Krijger GC, Lafeber FPJG, Beekman FJ, Mastbergen SC, Weinans H (2018). Imaging of folate receptor expressing macrophages in the rat groove model of osteoarthritis: Using a New DOTA-Folate Conjugate. Cartilage..

[54] Pippenger BE, Duhr R, Muraro MG, Pagenstert GI, Hügle T, Geurts J (2015). Multicolor flow cytometry-based cellular phenotyping identifies osteoprogenitors and inflammatory cells in the osteoarthritic subchondral bone marrow compartment. Osteoarthritis Cartilage..

[55] Siebelt M, van der Windt AE, Groen HC, Sandker M, Waarsing JH, Müller C, de Jong M, Jahr H, Weinans H (2014). FK506 protects against articular cartilage collagenous extra-cellular matrix degradation. Osteoarthritis Cartilage..

