# Supplementary material for: Clinical-grade human dental pulp stem cells suppressed the activation of osteoarthritic macrophages and attenuated cartilaginous damage in a rabbit osteoarthritis model
Source: Stem Cell Res Ther. 2021 May 1;12:260. doi: 10.1186/s13287-021-02353-2 (PMC8088312; doi:10.1186/s13287-021-02353-2)
Supplement: Supplementary file 1 — Additional file 1: Table S1. [file 13287_2021_2353_MOESM1_ESM.docx]

Table S1. Demographic, clinical, and imaging characteristics of the sample population

| No. of  patients | Age (y) | sex | BMI (kg/m^2^) | Symptom duration (years) | MOAKS Synovitis/effusion |
| --- | --- | --- | --- | --- | --- |
| 1 | 70-79 | 2 | 24 | 8 | 3 |
| 2 | 60-69 | 1 | 25 | 9 | 2 |
| 3 | 70-79 | 1 | 26 | 11 | 3 |
| 4 | 60-69 | 2 | 24 | 9 | 2 |
| 5 | 50-59 | 1 | 25 | 3 | 2 |
| 6 | 50-59 | 1 | 25 | 4 | 2 |
| 7 | 50-59 | 1 | 30 | 8 | 2 |
| 8 | 50-59 | 1 | 23 | 5 | 2 |
| 9 | 50-59 | 2 | 24 | 4 | 2 |
| 10 | 50-59 | 1 | 27 | 7 | 2 |

*BMI, Body Mass Index, MOAKS, MRI Osteoarthritis Knee Score, M, Male, F, Female*
