# Supplementary material for: Clinical-grade human dental pulp stem cells suppressed the activation of osteoarthritic macrophages and attenuated cartilaginous damage in a rabbit osteoarthritis model
Source: Stem Cell Res Ther. 2021 May 1;12:260. doi: 10.1186/s13287-021-02353-2 (PMC8088312; doi:10.1186/s13287-021-02353-2)
Supplement: Supplementary file 2 — Additional file 2: Table S2–S4. [file 13287_2021_2353_MOESM2_ESM.docx]

Take cDNA for RT-qPCR reaction, add sample according to the ratio in Table S2, immediately centrifuge and mix well, and amplify according to the conditions in Table S3, perform 40 cycles. The Melt Curve Stage is automatically set by the instrument, as shown in Table S4.

Table S2

| Reagent | Usage |
| --- | --- |
| DNA template | 0.2ul |
| Forward primer（10uM） | 0.4ul |
| Reverse primer（10uM） | 0.4ul |
| 2×realstar green fast mixture with RoxⅡ | 10ul |
| RNase-free water | Add to 20ul |

Table S3

| Temperature | Time |
| --- | --- |
| 95°C | 2min |
| 95°C | 15sec |
| 60°C | 20sec |
| 72°C | 30sec |

Perform 40 cycles in the order of 95°C , 60°C and 72°C.

Table S4

| Temperature | Time |
| --- | --- |
| 95°C | 15sec |
| 60°C | 1min |
| 95°C | 15sec |
| 60°C | 15sec |
