# Supplementary material for: Clinical-grade human dental pulp stem cells suppressed the activation of osteoarthritic macrophages and attenuated cartilaginous damage in a rabbit osteoarthritis model
Source: Stem Cell Res Ther. 2021 May 1;12:260. doi: 10.1186/s13287-021-02353-2 (PMC8088312; doi:10.1186/s13287-021-02353-2)
Supplement: Supplementary file 3 — Additional file 3: Table S5. [file 13287_2021_2353_MOESM3_ESM.doc]

| Genes | primer sequences | Products(bp) |
| --- | --- | --- |
| GADPH  TNF-α  IL-10  IL-12b | forward, 5′- TCTGACTTCAACAGCGACACC -3′  reverse, 5′- GTTGCTGTAGCCAAATTCGTT -3′  forward, 5′- GAGGCCAAGCCCTGGTATG-3′  reverse, 5′- CGGGCCGATTGATCTCAGC-3′  forward, 5′-ACCAAGACCCAGACATCAA-3′  reverse, 5′- CATTCTTCACCTGCTCCAC-3′  forward, 5′- TCGGCAGGTGGAGGTCAGC-3′  reverse, 5′- CGCAGAATGTCAGGGAGAAGTAGG -3′ | 190  91  137  77 |

**Table S5: Primer sequences**
