# Supplementary figures and images for: Clinical-grade human dental pulp stem cells suppressed the activation of osteoarthritic macrophages and attenuated cartilaginous damage in a rabbit osteoarthritis model
Source: Stem Cell Res Ther. 2021 May 1;12:260. doi: 10.1186/s13287-021-02353-2 (PMC8088312; doi:10.1186/s13287-021-02353-2)

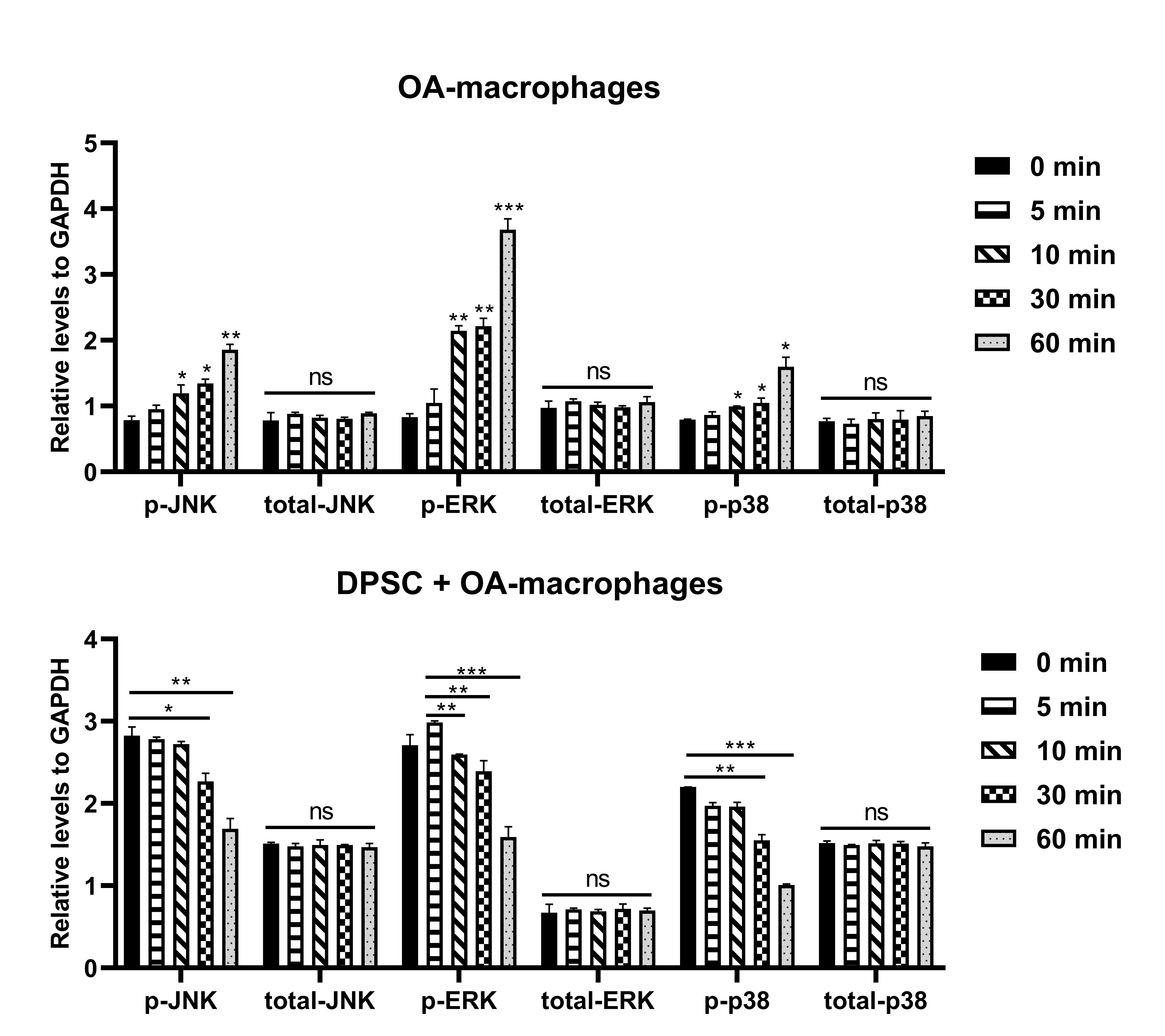

Supplement: Supplementary file 4 — Additional file 4: Figure S1. The MAPK pathways are closely involved in OA macrophage activation. The OASF activated MAPK pathway in OA macrophages in a time dependent manner. The conditional medium from hDPSCs significantly suppressed the activation in OA macrophages induced by OASF. The data are generated from western-blotting data from Fig. 4a by using greyscale software Fiji. *, P<0.05, **, P<0.01, ***, P<0.001. OASF: Synovial fluid from OA patients; OA: osteoarthritis. [file 13287_2021_2353_MOESM4_ESM.jpg]
